# Current Developments on the Role of α_1_-Adrenergic Receptors in Cognition, Cardioprotection, and Metabolism

**DOI:** 10.3389/fcell.2021.652152

**Published:** 2021-05-25

**Authors:** Dianne M. Perez

**Affiliations:** The Lerner Research Institute, The Cleveland Clinic Foundation, Cleveland, OH, United States

**Keywords:** adrenergic receptor, G-protein coupled receptor, cognition, cardioprotection, metabolism

## Abstract

The α_1_-adrenergic receptors (ARs) are G-protein coupled receptors that bind the endogenous catecholamines, norepinephrine, and epinephrine. They play a key role in the regulation of the sympathetic nervous system along with β and α_2_-AR family members. While all of the adrenergic receptors bind with similar affinity to the catecholamines, they can regulate different physiologies and pathophysiologies in the body because they couple to different G-proteins and signal transduction pathways, commonly in opposition to one another. While α_1_-AR subtypes (α_1A_, α_1B_, α_1C_) have long been known to be primary regulators of vascular smooth muscle contraction, blood pressure, and cardiac hypertrophy, their role in neurotransmission, improving cognition, protecting the heart during ischemia and failure, and regulating whole body and organ metabolism are not well known and are more recent developments. These advancements have been made possible through the development of transgenic and knockout mouse models and more selective ligands to advance their research. Here, we will review the recent literature to provide new insights into these physiological functions and possible use as a therapeutic target.

## Introduction

α_1_-Adrenergic receptors (ARs) regulate the sympathetic nervous system by binding and transducing the effects of the endogenous catecholamines, epinephrine, and norepinephrine (Graham and Lanier, 1986). ARs are members of the G-protein-coupled receptor (GPCR) superfamily and are composed of nine adrenergic receptor subtypes (α_1A_, α_1B_, α_1D_, α_2A_, α_2B_, α_2C_, β_1_, β_2_, and β_3_) from the three distinct families (α_1_, α_2_, β) which are activated by the same catecholamines and are related as paralogs.

The α_1_-AR subtype cDNAs were cloned in the late 1980s and early 1990s (Cotecchia et al., 1988; Schwinn et al., 1990; Lomasney et al., 1991; Perez et al., 1991, 1994; Laz et al., 1994). They have distinct pharmacological properties which helped to determine their classification and characterization. Before the cloning of the receptors, α_1_-ARs were already subdivided into the α_1A_- and α_1B_-AR subtypes based upon radioligand binding data in various tissues which showed two-site competition binding curves to the antagonists WB4101 and phentolamine. The α_1A_-AR subtype was defined as having a 10–100-fold higher binding affinity for these two antagonists while the α_1B_-AR subtype was defined as having the weaker binding affinity (Morrow and Creese, 1986). The α_1C_-AR designation is missing from the α_1_-AR subtype lineage because of a misclassification early on in the cloning of the receptors^1^.

α_1_-ARs are mainly coupled to the heterotrimeric Gq/11 (Gαq) family of G-proteins to activate phospholipase Cβ1 (PLCβ1), resulting in the hydrolysis of membrane-bound phosphatidylinositol 4,5-bisphosphate and the cytosolic release of inositol triphosphate (IP3) and diacylglycerol (DAG) (Piascik and Perez, 2001; Table 1). The IP3 plays a key role in calcium regulation by binding to IP3 receptors located on the endoplasmic reticulum resulting in calcium channel opening and the release of intracellular calcium. The DAG activates protein kinase C (PKC) which can phosphorylate many other types of proteins and signals downstream in the signaling cascade. There are also reports that α_1_-ARs can couple to G_i_ G-proteins under overexpressed conditions or in certain cell lines (Akhter et al., 1997; Melien et al., 2000; Snabaitis et al., 2005) but this has not been shown to occur *in vivo.* α_1_-ARs can also signal through G-protein-independent mechanisms involving β-arrestins which act as scaffolds to recruit and activate other second messengers such as ERK 1/2, p38, and Src (Perez-Aso et al., 2013; Segura et al., 2013). α_1_-ARs can also couple to phospholipase A_2_ and calcium channels though this may not be direct coupling (Perez et al., 1993).

**TABLE 1:** Properties of the α_1_-AR subtypes.

Subtype	α_1A_	α_1B_	α_1D_
Signal transduction	G_q_/G_11_/PLC/PKC/DAG/IP3/Ca^+2^ RGS2	G_q_/G_11_/PLC/PKC/DAG/IP3/Ca^+2^	G_q_/G_11_/PLC/PKC/DAG/IP3/Ca^+2^
Selective Agonists	A61603, cirazoline	None	None
Selective Antagonists	Niguldipine, 5-Methylurapidil,	None	BMY-7378
Allosteric	Amilorides (NAMs) 9-aminoacridine (NAM)	Conopeptide rho-TIA (NAM) 9-aminoacridine (NAM)	None
Tissue distribution	Hippocampus, amygdala, cerebral cortex, neural stem and progenitor cells, interneurons, hypothalamus, myocyte, smooth muscle, vascular, mesenteric arteries	Cerebral cortex, myocyte, smooth muscle, vascular	Reticular thalamic nuclei, hippocampus, spinal cord, aorta, smooth muscle, vascular, coronary arteries
Physiological function	Cognition, neurogenesis, LTP, spatial memory, blood pressure, positive inotropy, contraction smooth muscle, blood pressure, cardiac hypertrophy, cardiac adaptive, cardiac ischemic protection, glucose uptake (all tissues), glycolysis (cardiac, adipocytes, skeletal muscle), glucose tolerance, whole body fatty acid oxidation.	Memory consolidation, fear-motivated exploration, spatial learning-novelty, contraction smooth muscle, blood pressure, negative inotropy, cardiac hypertrophy, cardiac maladaptive, baroreflex, glucose uptake (non-cardiac tissues), glycolysis (adipocytes, skeletal muscle), glucose tolerance, whole body fatty acid oxidation.	Contraction smooth muscle, contraction-mesenteric beds, blood pressure.

While the α_1_-AR subtypes display differences in internalization resulting in spatio-temporal changes in signaling (Stanasila et al., 2008; Perez-Aso et al., 2013; Segura et al., 2013), there is some evidence that the α_1_-AR subtypes differentially couple to different signaling proteins, such as Regulators of G-protein Signaling (RGS) (Hague et al., 2005). These G-protein modulators can interact with the alpha subunits of large G-proteins to increase the rate of GTP hydrolysis and to stop the receptor signaling process. RGS2 can directly bind to the third intracellular loop of the α_1A_-AR to inhibit its signaling process but does not bind at the α_1B_- or α_1D_-AR subtypes (Hague et al., 2005). As RGS2 plays a prominent role in regulating GPCR cardiovascular functions (Tang et al., 2003; Zou et al., 2006) and GPCR G_11_ signaling pathways (Cunningham et al., 2001), α_1A_-AR coupling to RGS2 may regulate many of its subtype-specific functions. Another way that α_1_-ARs create differential signaling pathways is through biased agonism (Wootten et al., 2018). Cirazoline or A61603, imidazolines which are α_1A_-AR selective agonists, can bias the receptor toward cAMP signaling rather than Ca^+2^ release or ERK phosphorylation (Evans et al., 2011; da Silva et al., 2017) or can enhance the α_1A_-AR desensitization and internalization process (Akinaga et al., 2013) leading to differential coupling to β-arrestin-mediated signaling.

## Transgenic and Knockout Mouse Models

Due to the lack of sufficiently selective pharmacological agents to use in order to distinguish subtype-specific effects, a number of transgenic and knockout (KO) mouse models were developed that were used to determine long-term *in vivo* stimulatory or inhibitory effects of the α_1_-AR subtypes on physiology and pathophysiology (Table 2). KOs of the α_1A_-AR (Rokosh and Simpson, 2002; Zhang et al., 2020), α_1B_-AR (Cavalli et al., 1997), and α_1D_-AR (Tanoue et al., 2002) were developed using traditional insertion of the β-galactosidase or neomycin resistance gene in place of the first exon of the receptor. Recently, a cardiac-conditional KO of the α_1A_-AR was developed (Zhang et al., 2020). There is also a double KO model created by mating together the α_1A_ and α_1B_-AR KO mice (O’Connell et al., 2003) and a triple KO of all three subtypes (Sanbe et al., 2007). Transgenic mice overexpressing α_1_-ARs were designed to either target to the myocyte using the α myosin heavy chain promoter to drive only cardiac expression of wild-type (WT) or constitutively active mutations (CAMs) in the receptor (Milano et al., 1994; Grupp et al., 1998; Eckhart et al., 2000; Lin et al., 2001) or used CAMs in the receptors that were driven by large fragments of the endogenous mouse promoters to generate systemic expression (Zuscik et al., 2000, 2001; Ross et al., 2003; Rorabaugh et al., 2005). The systemic expression of the CAMs also allows assessment of cardiovascular effects due to chronic α_1_-AR expression outside of the myocyte as well as in the brain or other organ systems. There is also only mild overexpression of the receptor in the heart and brain (2–3 fold) and throughout the body in using the endogenous promoters as compared to using the α myosin heavy chain promoter which caused very high amounts of receptor overexpression, often exceeding 100-fold. The use of CAMs instead of the WT receptor results in continuously activated receptors that do not need an agonist to be present and can be representative of a chronically stimulated condition, but this is still debated. In both overexpressed and KO mouse models, there is always the possibility of changes in the expression of other genes and receptors in compensation or as a result of additional insertion or deletion of genetic material, a widespread phenomenon that is hard to decipher and under reported (El-Brolosy and Stainier, 2017). Recognizing these limitations and seeing if general phenotypes repeat in the various mouse models of particular receptor subtypes is suggested. These different types of mouse models will be referred to throughout this review.

**TABLE 2:** Genetic animal models of the α_1_-AR subtypes.

Animal model	Genotype	Cognitive phenotype	Cardiac phenotype	Metabolic phenotype	References
α_1A_-AR	CAM, systemic overexpression expression (2–3 fold)	Increased spatial memory, learning, LTP, paired pulse, neurogenesis	Adaptive-ischemic preconditioning, increased contractility, no changes in BP	Higher whole-body FAO, increased glucose uptake in cardiac and other tissues, cardiac glucose oxidation, glucose tolerance, leptin secretion	Ross et al., 2003; Rorabaugh et al., 2005; Gupta et al., 2009; Shi et al., 2016, 2017; Papay and Perez, 2020; Perez, 2021
α_1A_-AR	αMHC, heart-targeted overexpression (66-fold)		Adaptive-increased inotropy, protects after TAC and MI, no hypertrophy, angiogenesis		Lin et al., 2001; Du et al., 2004, 2006; Zhao et al., 2015
α_1A_-AR	αMHC, heart-targeted overexpression (170-fold)		Maladaptive-increased mortality, fibrosis in aged mice		Chaulet et al., 2006
α_1A_-AR (rats)	αMHC, heart-targeted overexpression		Adaptive-protects against MI, ischemic preconditioning		Zhao et al., 2012, 2015
α_1B_-AR	αMHC, heart-targeted overexpression (26 and 46-fold)		Maladaptive-negative inotropy, dilated cardiomyopathy, no hypertrophy		Akhter et al., 1997; Grupp et al., 1998; Lemire et al., 2001
α_1B_-AR	CAM, αMHC, heart-targeted overexpression (3-fold)		Maladaptive-hypertrophy, increased progression to HF, no preconditioning		Milano et al., 1994; Gao et al., 2000; Wang et al., 2000
α_1B_-AR	CAM, systemic overexpression expression (2-3 fold)	Autonomic failure; Parkinson’s Disease Plus neurodegeneration	Maladaptive-negative inotropy, hypertrophy in older mice, fibrosis, hypotension	Higher whole-body FAO, increased glucose tolerance and uptake in non-cardiac tissues, leptin secretion	Zuscik et al., 2000, 2001; Ross et al., 2003; Papay et al., 2013; Shi et al., 2016, 2017
α_1AB_-AR	CAM double systemic overexpression		No basal hypertrophy but induced when either α_1A_- or α_1B_-ARs are individually stimulated		Papay et al., 2013
α_1A_-AR	KO	Poor cognitive behavior	Maladaptive-increased pathology after MI, normal heart size	Higher whole-body carbohydrate oxidation, decreased cardiac glucose uptake, glucose intolerance	Doze et al., 2011; Shi et al., 2016, 2017; Yeh et al., 2017
α_1A_-AR	Conditional heart-targeted KO		Maladaptive-increased mortality; increased pathology after MI		Zhang et al., 2020
α_1B_-AR	KO	Locomotor, decreased addiction, memory consolidation, novelty/fear memory	No changes in basal BP, decreased induced BP; loss of NE-induced hypertrophy, decreased baroreflex response	Insulin resistance, higher whole-body carbohydrate oxidation, glucose intolerance and decreased glucose uptake in non-cardiac tissues and leptin secretion	Knauber and Müller, 2000a; Spreng et al., 2001; Drouin et al., 2002; Vecchione et al., 2002; Auclair et al., 2004; Burcelin et al., 2004; Townsend et al., 2004
α_1D_-AR	KO	Decreased locomotion, attention	Decrease basal and induced BP		Sadalge et al., 2003; Hosoda et al., 2005
α_1A/B_-AR	Double KO		Maladaptive- loss of heart growth, decreased survival and contractility after TAC, fibrosis, apoptosis		McCloskey et al., 2003; O’Connell et al., 2003; Turnbull et al., 2003
α_1A/B/D_-AR	Triple KO		Hypotension		Sanbe et al., 2007

*BP, blood pressure; CAM, constitutively active mutation(s); FAO, fatty acid oxidation; HF, heart failure; KO, knockout; LTP, long-term potentiation; MHC, myosin heavy chain promoter; MI, myocardial infarction; TAC, transverse aortic constriction.*

## Cognition

### Localization in the Brain

The expression of the specific α_1_-AR subtypes in the brain was previously difficult to determine because of the lack of high avidity antibodies to the α_1_-ARs (Jensen et al., 2009c; Böhmer et al., 2014). Initial autoradiography studies used non-selective radiolabels that could not distinguish between the α_1_-ARs subtypes but did demonstrate high abundance throughout the rat brain (Unnerstall et al., 1985). Eventually, more specific and sensitive techniques were developed to determine the α_1_-AR subtype localization in the brain such as using the full-length cDNA sequence of the α_1A_-AR in hybridization studies (Domyancic and Morilak, 1997) or transgenic and knock-out (KO) mouse models of the α_1_-AR subtypes with the α_1_-ARs tagged with endogenous promoter-driven expression of EGFP or use of the β-galactosidase gene to KO the receptor (Papay et al., 2004, 2006). Using these approaches, the α_1A_- and α_1B_-ARs were shown to be expressed in similar areas of the brain, but the relative expression was different (Papay et al., 2004, 2006). The α_1A_-AR subtype was more noticeably expressed in the cognitive areas such as the hippocampus, amygdala, and particular cortical areas (Table 1; Papay et al., 2006), while the α_1B_-AR appeared more prominent throughout the cortex and thalamus (Drouin et al., 2002; Papay et al., 2004). The α_1A_-AR subtype was also more prominently expressed in neural progenitors and stem cells (Papay et al., 2006; Gupta et al., 2009). Using long sequences of antisense to the α_1D_-AR to assess brain localization, the α_1D_-AR although of low overall abundance, was present in the reticular thalamic nuclei, hippocampus, cortex and spinal cord (Harasawa et al., 2003). Using the α_1_-AR KO mice and comparing the total amount of α_1_-AR radioligand receptor binding to normal wild-type mice, it was concluded that the brain contains the highest amount of the α_1A_-AR subtype at ∼55% (Rokosh and Simpson, 2002), followed by the α_1B_-AR at 35% (Cavalli et al., 1997) but only 10% of the total α_1_-AR pool for the α_1D_-AR subtype (Tanoue et al., 2002; Sadalge et al., 2003).

The localization of the α_1_-ARs in the brain may have some species variation (Palacios et al., 1987; Zilles et al., 1991), but the cognitive areas appear similar in humans with high expression in the hippocampus and prefrontal cortex and the lowest expression in the caudate and putamen (Shimohama et al., 1986; Szot et al., 2005). The α_1A_-AR subtype appears to be prominent in expression in the hippocampus as assessed by RNA (Szot et al., 2005), single cell PCR (Hillman et al., 2005), protein localization using the EGFP-tagged transgenics (Papay et al., 2006) and functionally by regulating the CA1 hippocampal interneurons (Jurgens et al., 2009). In addition, the α_1A_-AR subtype regulated adult neurogenesis in the mouse subgranular and subventricular zones (Gupta et al., 2009; Jurgens et al., 2009; Collette et al., 2010) as assessed by increased BrdU incorporation and co-localization studies of EGFP-tagged α_1A_-ARs with stem cell and neural progenitor markers (Table 2). In addition, when normal WT mice were given the α_1A_-AR selective agonist, cirazoline, they also displayed increased neurogenesis (Gupta et al., 2009). The regulation of neurogenesis by the α_1A_-AR and its regulation of hippocampal function and translation to human brain domains may potentially play a therapeutic role to increase synaptic plasticity and cognition in diseases of dementia.

### General Cognition

The α_1_-ARs have been previously associated with general roles in learning and memory functions (Sirviö and MacDonald, 1999) but these studies were not well characterized nor assigned to specific AR subtypes because of the lack of subtype-specific ligands. A few early studies suggested that α_1_-AR stimulation inhibits memory functions in monkeys (Arnsten and Jentsch, 1997; Mao et al., 1999) or in chickens (Gibbs and Summers, 2001) but used very low replicates, very high concentrations of ligands rendering them non-selective or attributed to species variation. However, as will be discussed, most of the recent studies indicate that α_1_-AR stimulation increases various types of memory in both formation and storage.

### Long-Term Potentiation

Long-term potentiation (LTP) is a type of long-lasting synaptic plasticity that increases the strength of synaptic transmission over a long period of time (i.e., mins-hours) (Hopkins and Johnston, 1984; Kandel, 2001). LTP is considered a major mechanism of learning and memory, particularly in the hippocampus (Bliss and Collingridge, 1993). α_1_-AR stimulation can induce LTP in the hippocampus (Izumi and Zorumski, 1999; Sirviö and MacDonald, 1999; Lv et al., 2016) and there is one report in the neocortex (Pankratov and Lalo, 2015) which is also a center for neuronal spatial and recognition memory (Vann and Albasser, 2011). Interestingly, the α_1_-ARs can also stimulate ATP release on astrocytes to induce LTP via ATP receptors on the pyramidal neurons in the neocortex, suggesting that glial cell regulation by α_1_-ARs may also be involved in memory formation. Glia communicate through calcium signaling to neurons, causing the release of ATP and its subsequent increase in synaptic plasticity and LTP (Pascual et al., 2005). LTP stimulation by α_1_-ARs may be α_1A_-AR-specific as the CAM α_1A_-AR transgenic mice significantly increased LTP at hippocampal synapses (Doze et al., 2011; Table 2). The CAM α_1A_-AR mice also increased cognitive scores in a series of behavioral tests while the α_1A_-AR KO mice performed poorly compared to normal controls (Doze et al., 2011). The α_1A_-AR selective agonist, cirazoline also increased cognitive scores in normal mice when administered for 2 months. While the α_1B_-AR KO mice had impaired cognition in some behavior tests (Knauber and Müller, 2000a, b; Spreng et al., 2001), there was no assessment of effects of α_1B_- or α_1D_-AR KO on LTP.

Long-term depression (LTD) is also a form of long-term synaptic plasticity that can contribute to cognitive functions by increasing the flexibility of the synapse to store information (Heynen et al., 1996), such as remembering the exposure to novel objects (Manahan-Vaughan and Braunewell, 1999). Novelty exposure can reverse LTP in the hippocampus (Xu et al., 1998), suggesting a correlation between LTD and LTP that may impart different forms of synaptic information during spatial learning (Kemp and Manahan-Vaughan, 2004). There are reports that α_1_-AR mediated LTD required co-activation with a number of partners such as β-ARs (Katsuki et al., 1997), NMDA (Scheiderer et al., 2004) and the M1 muscarinic receptor (Scheiderer et al., 2008). α_1_-ARs have been shown to induce LTD at excitatory CA3–CA1 synapses in the rat hippocampus (Dyer-Reaves et al., 2019) through ERK signaling in the pyramidal neurons (Vanhoose et al., 2002; Scheiderer et al., 2008) and had characteristics of a novel form of synaptic plasticity (Hebb, 1949). However, there is no evidence of which α_1_-AR subtype(s) mediate LTD. This Hebbian LTD requires coincident presynaptic and postsynaptic NMDAR activity (Scheiderer et al., 2004) and is different and independent of the “classical” LTD which is induced by low frequency synaptic stimulation that is repetitive (Mulkey and Malenka, 1992). The mechanism of the Hebbian LTD also involves postsynaptic activation of the α_1_-AR as the paired pulse facilitation ratio did not change (Scheiderer et al., 2004). Paired pulse facilitation is a measurement of synaptic enhancement observed under a short period of time (i.e., milliseconds). For a pulse facilitation effect, a second evoked excitatory postsynaptic potential is increased when it follows immediately after a first evoked excitatory postsynaptic potential (Foster and McNaughton, 1991) and is used as evidence of an increase in the probability of neurotransmitter release. Increases in paired pulse facilitation that occur with LTP suggest a presynaptic mechanism (Schultz et al., 1994), because potentiated presynaptic neurons must increase neurotransmitter release.

### Spatial Memory

The hippocampus also regulates spatial and associative learning functions (Mahmoodi et al., 2010) in addition to long-term memory functions. α_1_-AR blockage using the α_1_-AR antagonist prazosin in the hippocampus demonstrated impaired spatial learning (Petrasek et al., 2010) while stimulation of the α_1_-AR improved spatial memory (Puumala et al., 1998; Torkaman-Boutorabi et al., 2014). Transgenic mice overexpressing CAM α_1A_-ARs, or WT mice given the α_1A_-AR selective agonist cirazoline, displayed increased learning and memory using several spatial memory behavioral tests such as the Barnes, dry multi-T, and Morris water mazes (Doze et al., 2011), while α_1A_-AR KO mice showed decreased learning and memory compared to normal controls in the same cognitive tests (Doze et al., 2011; Collette et al., 2014; Table 2). The α_1B_-AR KO mice also had impaired spatial learning to novelty and exploration (Spreng et al., 2001) and a decrease in non-spatial memory functions such as memory consolidation, fear-motivated exploration (Knauber and Müller, 2000a), and short and long-term latency in a passive avoidance test (Knauber and Müller, 2000b). α_1D_-AR KO mice did not show changes in several different behavioral cognitive tests (Sadalge et al., 2003) but did show changes in locomotion and attention (Mishima et al., 2004). Together with enhancement of LTP and paired pulse facilitation (a type of short-term synaptic plasticity) in the CAM α_1A_-AR transgenic mice (Doze et al., 2011), these studies suggest that the α_1A_- and perhaps the α_1B_-AR to a lesser degree but not the α_1D_-AR are involved in spatial learning and memory processes.

### Spatial Working Memory

Spatial working memory involves executive-type or motivational-related types of memory and relies more on the prefrontal cortex than the hippocampus as the task is more complex (Robbins, 1996). α_1_-AR stimulation increases while α_1_-AR blockade inhibits working memory (Pussinen et al., 1997; Puumala et al., 1998; Lapiz and Morilak, 2006; Hvoslef-Eide et al., 2015) by promoting both focused and flexible attention (Berridge et al., 2012; Berridge and Spencer, 2016). There is also an improvement in working memory with the cognitive-enhancing, wake-promoting neurochemical modafinil that is hypothesized to be mediated by α_1_-ARs since effects are blocked by prazosin (Duteil et al., 1990; Stone et al., 2002; Winder-Rhodes et al., 2010).

α_1_-ARs regulate spatial working memory through the release of glutamate in the prefrontal cortex due to a sustained excitatory effect on the pyramidal neurons increasing synaptic plasticity (Marek and Aghajanian, 1999; Zhang et al., 2013). When the ventral hippocampus was lesioned *in vivo* and α_1_-AR function was impaired, there was a decrease in glutamatergic synaptic plasticity within the prefrontal cortex which caused memory and learning dysfunction (Bhardwaj et al., 2014). Glutamatergic synaptic plasticity mediated through α_1_-ARs signals through PKC-dependent pathways in various cortical areas (Mouradian et al., 1991; Marek and Aghajanian, 1996; Chen et al., 2006; Kobayashi et al., 2008; Velásquez-Martinez et al., 2012; Luo et al., 2014, Luo et al., 2015a, b) and may require the co-signaling from both glutamate and the N-type Ca^2+^ channels (Luo et al., 2015a). PKC can increase synaptic plasticity and associated memory processes through the phosphorylation of synaptic proteins or enhancing the sensitivity to calcium which promotes the exocytosis of the synaptic vesicles, increasing neurotransmitter release (Shimazaki et al., 1996; Stevens and Sullivan, 1998; Hilfiker and Augustine, 1999; Wu and Wu, 2001).

Besides glutamatergic mechanisms, the disruption of GABAergic transmission in the prefrontal cortex can also cause a decrease in working memory (Enomoto et al., 2011; Bañuelos et al., 2014). α_1_-AR stimulation in the medial prefrontal cortex inhibits the inwardly rectifying potassium channels (Kirs) located on the interneuron, leading to depolarization and an increased calcium influx through calcium channels resulting in increased GABAergic transmission onto the pyramidal neurons (Luo et al., 2015b). The excitation can be enhanced when the α_1_-ARs stimulation is facilitated by postsynaptic α_2_-ARs decreasing the hyperpolarization of cyclic nucleotide-gated cation channels (Zhang et al., 2013). Therefore, α_1_-ARs may work to improve spatial working memory through both glutamatergic and GABAergic mechanisms which suggests that α_1_-AR agonists could be used to target enhancement of spatial working memory.

### Memory Consolidation

α_1_-AR activation can enhance memory recall and consolidation. The process of memory consolidation changes recent and labile memories into long-lasting ones. The process starts in the hippocampus but as time passes and the memory is reorganized, the long-lasting memory is then distributed in the neocortex (Squire et al., 2015). The α_1_-AR antagonist, prazosin, blocked the norepinephrine-facilitated reconsolidation of memory during fear conditioning (Gazarini et al., 2013) and the consolidation of both short-term and intermediate-term memory in chickens (Gibbs and Bowser, 2010). The mechanism for α_1_-ARs to consolidate memories was suggested to be mediated through an increase in free cytosolic calcium in astrocytes as effects were blocked with glycolytic inhibitors (Gibbs and Bowser, 2010). Astrocytes, unlike neurons, mediate learning and memory utilizing glycogenolysis, which the astrocyte needs for the synthesis of glutamate (Gibbs et al., 2008; Newman et al., 2011).

The basolateral nucleus of the amygdala (BLA) can also be involved in the storage and consolidation of memory (Ferry and McGaugh, 2000). As cAMP signaling is mainly involved in mediating the effects of norepinephrine on memory consolidation, the β-ARs were previously considered the main AR to transduce those effects (Ikegaya et al., 1997; Ferry and McGaugh, 2000; Ferry and Quirarte, 2012). However, both β- and α_1_-ARs may be needed together to mediate memory storage in the BLA. The stimulation of cAMP through a β-AR agonist in the BLA can be blocked with an α_1_-AR antagonist and memory storage is increased with use of a synthetic cAMP analog (Ferry et al., 1999a, b). Similarly, stimulation of α_1_-ARs can potentiate β-AR-mediated cAMP formation in the BLA to enhance memory storage (Ferry et al., 1999a, b). α_1B_-AR KO mice had a decrease in latency in the passive avoidance test suggesting deficits in memory consolidation *in vivo* (Knauber and Müller, 2000b; Table 2). Research performed in amnesia patients developed the concept of memory consolidation as time was needed for this process to occur and greater memory deficits were seen in retrograde amnesia patients with loss of information from recent memory (Brown, 2002). α_1_-AR stimulation can reverse cannabinoid-induced (Moshfegh et al., 2011) and scopolamine-induced amnesia (Azami et al., 2010) and enhance recall when α_1_-AR agonists were administered before electroconvulsive shocks (Anand et al., 2001).

### Dementia-Related Diseases

α_1_-AR functions may change and contribute to the aging process in the loss of memory function. α_1_-AR protein is increased in the aging mouse brain and with improved learning, supporting a role for these receptors in age-related cognitive decline (Knauber and Müller, 2000b). In patients suffering from Alzheimer’s Disease (AD), α_1_-AR protein and mRNA is reduced in the prefrontal cortex (Shimohama et al., 1986; Kalaria, 1989; Szot et al., 2007). The mRNA levels of the α_1A_-AR were significantly decreased in the prefrontal cortex with AD with no changes in the mRNA of the α_2_-AR (Szot et al., 2007). There is also an α_1A_-AR polymorphism that associates with AD (Hong et al., 2001). Decreases in spatial memory that are due to the aging process were improved in rats when the α_1_-AR was stimulated (Riekkinen et al., 1997).

The 3xTG (Transgenic) is a widely used AD mouse model that contains three genetic mutations associated with familial AD (APP Swedish, MAPT P301L, and PSEN1 M146V) (Oddo et al., 2003). This AD mouse model displays β-amyloid deposits, tau immunoreactivity, cognitive impairment, and decreases in LTP and basal synaptic transmission (Oddo et al., 2003). When the 3xTG AD mouse model was given a selective α_1A_-AR positive allosteric modulator, spatial memory as assessed in the Barnes maze was improved along with LTP (Perez, 2021). These results suggest that selective agonists that increase α_1__A_-AR functions may be able to improve cognitive decline in AD.

Another cognitive disease is vascular dementia which is the second-most frequent form of dementia after AD. α_1_-AR autoantibodies with agonistic function were found in 50% of people with dementia (Karczewski et al., 2010, 2012, 2018; Hempel et al., 2016; Thyrian et al., 2018). While these agonistic autoantibodies may also cause vascular damage, shown for several neurotransmitters (Wu and Li, 2016), one interpretation of the data consistent with the role of the α_1A_-AR in improving cognition, but also speculative, is that they may develop during dementia to compensate for the loss in receptor density as documented by Shimohama et al. (1986) and Szot et al. (2007).

## Cardioprotection

The heart expresses both the α_1A_ and α_1B_-AR subtypes with relative expression levels depending upon the species (Steinfath et al., 1992; Michel et al., 1994; Jensen et al., 2009a). The α_1D_-AR is weakly expressed if at all in the myocyte (Price et al., 1994; Scofield et al., 1995) but is present in vascular smooth muscle, particularly in the coronary arteries, mesenteric beds and the aorta (Table 1; Hrometz et al., 1999; Gisbert et al., 2002; Chalothorn et al., 2003; Turnbull et al., 2003; Hosoda et al., 2005; Jensen et al., 2009b; Methven et al., 2009; Martínez-Salas et al., 2011). A KO mouse model of the α_1B_-AR was created with a human placental alkaline phosphatase inserted into the first exon to facilitate reporting (Myagmar et al., 2017). Using this new KO model and the conventional α_1A_-KO which has the β-galactosidase reporter, the authors report a heterogenous population of the α_1B_ and α_1A_-AR subtypes in the myocytes. The α_1B_ was present in all of the myocytes but the α_1A_ was present in only 60% of the myocytes and 20% of those had very high expression levels. This intermittent variable expression of the α_1A_-AR subtype was also observed in the mesenteric arteries in the α_1B/D_ double KO and in the transgenic systemically expressing α_1_-AR WT mice that were tagged with the green fluorescent protein (Papay et al., 2004; McGrath, 2015). Since this intermittent expression is only present in genetically altered mouse models, this suggests that intermittent expression may be an artifact. However, the current lack of highly avid α_1_-AR antibodies that can be used for *in vivo* localization (Jensen et al., 2009c; Böhmer et al., 2014), precludes using immunoassays to determine if intermittent expression is an artifact. A potential experiment that may confirm intermittent expression in a WT mouse would be to perform autoradiography with and without selective α_1_-AR blockers such as niguldipine to block the α_1A_-AR subtype.

It is generally accepted that α_1_-AR stimulation can regulate a positive inotropic response in the heart, although the response can be variable and display negative inotropy depending upon the species and the region in the heart analyzed (Endoh et al., 1991; Nishimaru et al., 2001; Endoh, 2016). The α_1A_- and not the α_1B_-AR is suggested to play a role in positive inotropy (Lin et al., 2001; Ross et al., 2003; Luo et al., 2007; Janssen et al., 2018). The systemically over-expressed CAM α_1B_-AR mice had no changes in basal cardiac parameters but had autonomic failure (Zuscik et al., 2001). The autonomic failure in the CAM α_1B_-AR mice indicated reduced circulating catecholamine levels, bradycardia, reproductive problems and weight loss. Together with the widespread neurodegeneration and a phenotype that was consistent with a Parkinson Disease plus syndrome, the basal hypotension seen in these mice was likely due to the autonomic failure rather than a direct effect on the ability to contract vascular smooth muscle. The CAM α_1B_-AR mice also had a negative inotropic response to phenylephrine (Ross et al., 2003). Radioligand binding analysis revealed that there was decreased α_1A_-AR density which was likely causing the negative inotropic effect (Ross et al., 2003). This functional antagonism of the positive inotropy of the α_1A_-AR by the α_1B_-AR was also found in a mouse model of right ventricular failure (Cowley et al., 2015). The heart-targeted WT α_1B_-AR also displayed negative inotropy (Grupp et al., 1998). In contrast, both the cardiac-targeted WT and systemically expressed CAM α_1A_-AR mediated a positive inotropic response in the mouse heart (Lin et al., 2001; Rorabaugh et al., 2005; Table 2). In human myocardium, the α_1A_-AR selective agonist, A61603, had a strong positive inotropic response representing about 70% of the β-AR response (Janssen et al., 2018).

### Heart Failure

In human heart failure, radioligand binding indicates that β_1_-ARs are downregulated (Bristow et al., 1982, 1986; Rockman et al., 2002) while α_1_-AR are either unchanged (Bristow et al., 1988; Jensen et al., 2009a) or decreased (Limas et al., 1989; Zhao et al., 1996; Fischer et al., 2008; Shi et al., 2013). MicroRNA-133 was found to be a key control in the downregulation of the β_1_-AR and several components of its signal transduction cascade in the heart (Castaldi et al., 2014), opening up new avenues of therapeutics in addition to β-blockers. Radioligand binding of human hearts with end-stage dilated cardiomyopathy versus non-failing controls revealed that while β_1_-ARs are downregulated as previously reported (Bristow et al., 1982, 1986), there was also a loss in the α_1A_-AR subtype receptor levels (Shi et al., 2013). The differences in these studies of the density of α_1_-ARs could be the severity of the heart failure (Limas et al., 1989), the level of sympathetic overdrive (Zhao et al., 1996) or the etiology of heart failure studied (ischemic versus non-ischemic) as α_1_-ARs are known to increase in density during ischemia (Corr et al., 1981; Maisel et al., 1987; Kurz et al., 1991) and could have masked the decrease in α_1A_-ARs during failure.

α_1_-ARs also can mediate cardiac hypertrophy, an increase in protein mass of the myocyte through an increase in protein synthesis which remodels the heart in response to various physiological and pathophysiological stimuli (Simpson, 1983; Fuller et al., 1990; Ikeda et al., 1991; Perez-Aso et al., 2013; Cotecchia et al., 2015). While both the α_1A_ and α_1B_-ARs are involved in hypertrophy, the α_1A_-AR seems better coupled to enhance hypertrophic signaling pathways. The α_1A_-AR agonist, A-61603, increased the size of the myocyte by increasing the rate of protein synthesis (Autelitano and Woodcock, 1998). The various transgenic mouse models showed variable degrees of cardiac hypertrophy but have never been as robust as seen in cell cultures (Table 2). Cardiac hypertrophy can be a normal physiological response which is adaptive and improves function while hypertrophy that is associated with fibrosis or apoptosis is maladaptive and can lead to heart failure. Both the α_1A_- and α_1B_-AR subtypes are required for physiological cardiac hypertrophy (O’Connell et al., 2003) as single KO do not have decreased heart size (Vecchione et al., 2002; Table 2). The systemic-expressing CAM α_1A_ displayed adaptive cardiac hypertrophy without increasing blood pressure (Papay et al., 2013). The heart-targeted CAM α_1B_ mouse induced hypertrophy (Milano et al., 1994) but displayed maladaptive remodeling after pressure overload (Wang et al., 2000). The systemically expressing CAM α_1B_ also induces cardiac hypertrophy (Zuscik et al., 2001) but was more pronounced when the mouse aged (Papay et al., 2013). A systemically expressing WT α_1B_-AR also displayed a lower degree of hypertrophy that only manifested in aged mice with fibrosis indicating a maladaptive cardiac hypertrophy (Zuscik et al., 2001). KO of the α_1B_-AR had a loss of NE-induced hypertrophy but not a decrease in heart size at birth (Vecchione et al., 2002). While a heart-targeted WT α_1B_ with high overexpression did not induce hypertrophy, it did induce a maladaptive dilated cardiomyopathy (Akhter et al., 1997; Grupp et al., 1998; Lemire et al., 2001). The α_1B_-AR has been suggested to regulate cardiac hypertrophy differently than the α_1A_-AR and the two AR subtypes may need to be co-activated to regulate hypertrophy (Papay et al., 2013). The CAM α_1A_-AR mice selectively secreted interleukin-6 (IL-6) and atrial naturietic factor while the CAM α_1B_-AR mice activated nuclear factor-kB (Papay et al., 2013). The α_1AB_-AR double KO mice also failed to develop hypertrophy when stimulated with IL-6 but WT mice developed hypertrophy when given IL-6. These hypertrophic signals were blocked in each mouse model and no increase in heart weight observed when the other AR was coactivated or when the two transgenic mouse models were crossbred, resulting in a CAM α_1A/B_-AR double transgenic mouse model (Papay et al., 2013). Hypertrophy became apparent in the CAM α_1AB_-AR double transgenic when either the α_1A_-AR or α_1B_-AR were independently stimulated (Papay et al., 2013). These results suggest that both the AR subtypes can increase hypertrophy through different signaling pathways. Increased α_1A_-AR signaling can induce an adaptive hypertrophy consistent with its postulated role of cardiac protection while increased α_1B_-AR signaling induces a maladaptive hypertrophy in the heart. These differences between adaptive versus maladaptive hypertrophy may be due to differences in α_1_-AR mediation of IL-6, ANF, and NF-kB signaling pathways.

### α_1A_-AR Mediated Protection in Heart Failure

It is postulated that selective α_1A_-AR stimulation may be a potential therapeutic in heart failure (Perez and Doze, 2011; Janssen et al., 2018) while α_1B_-AR stimulation, on the other hand, is maladaptive. This is evidenced by the heart-targeted WT α_1B_-AR mice induced dilated cardiomyopathy (Lemire et al., 2001) while heart-targeted CAM α_1B_-AR progressed to heart failure after pressure-overload (Wang et al., 2000; Table 2). In contrast, the heart-targeted WT α_1A_-AR mice were protected against pressure-overload induced heart failure (Du et al., 2004) or dysfunction due to myocardial infarction (Du et al., 2006) compared to non-transgenic controls. This mouse model also showed increased vascular endothelial growth factor-A expression which induced angiogenesis and resulted in increased capillary density and blood flow to the heart, postulated to be a contributing mechanism for cardioprotection (Zhao et al., 2015). This phenotype of induced angiogenesis could be reproduced when WT mice were given the α_1A_-AR agonist, A61603. A61603 or dabuzalgron also increased survival and prevented the damage due to the cardiotoxic agent, doxorubicin (Beak et al., 2017; Montgomery et al., 2017) and increased contraction in a mouse model of right heart failure (Cowley et al., 2015).

### Preconditioning and Ischemia

The high metabolic rate of the heart can cause the heart to be sensitive to the lack of oxygen (i.e., ischemia) resulting in injury to the muscle. α_1_-AR have long been known to mediate protective effects against ischemia or preconditioning in ischemia in several species (Banerjee et al., 1993; Kitakaze et al., 1994; Tsuchida et al., 1994; Salvi, 2001; Rorabaugh et al., 2005; Zhao et al., 2012; Nazari et al., 2019; Papay and Perez, 2020). In preconditioning, short periods of ischemia can stimulate signaling in the heart that protects the cardiac muscle from subsequent ischemic injury. The mechanism has been multi-faceted and attributed to PKC (Tsuchida et al., 1994; Mitchell et al., 1995; Rehring et al., 1996; Rorabaugh et al., 2005), mitochondrial potassium channels (Nazari et al., 2019), mitochondrial permeability transition pore (Naderi et al., 2010), 5′-nucleotidase activity (Tsuchida et al., 1994) or angiogenesis (Zhao et al., 2012). In recent studies, the ischemic protective effect of the α_1_-AR observed in primary cardiomyocytes was also proposed to be through the metabolic effects of glucose (Papay and Perez, 2020). Most models of ischemic preconditioning and particularly those by α_1_-ARs converge first on PKC, then diverge to other downstream effectors (Downey and Cohen, 1997; Simkhovich et al., 2013) and are postulated to also do so in the human heart (Speechly-Dick et al., 1995).

### α_1A_-AR Mediated Protection in Ischemia

The α_1A_-AR subtype has been shown to mediate the cardioprotective effects of α_1_-ARs in ischemic preconditioning. These studies have been performed in transgenic or KO mouse models as blocking one subtype is still not specific enough to perform with antagonists. The systemically expressed CAM α_1A_ mice were inherently preconditioned against ischemia while the CAM α_1B_ was not (Rorabaugh et al., 2005; Table 2). The heart-targeted CAM α_1B_-AR also did not show ischemic preconditioning (Gao et al., 2000). In corroboration, the heart-targeted WT α_1A_-AR transgenic rat exhibited preconditioning that appeared during the second window of protection that occurs days (and not minutes) after ischemia (Du et al., 2006; Zhao et al., 2012, 2015). There are also two reports that α_1B_-AR stimulation in WT mice can induce ischemic preconditioning involving PKC but used sensitivity to chloroethylclonidine as a criteria to block α_1B_-ARs selectively (Hu and Nattel, 1995; Gao et al., 2007). However, chloroethylclonidine was shown to not be selective against the α_1B_-AR but can block all the α_1_-AR subtypes (Xiao and Jeffries, 1998). Transgenic rats with myocyte-specific α_1A_-AR overexpression protected the heart from permanent coronary occlusion and during preconditioning (Zhao et al., 2012, 2015). The α_1A_-AR KO or conditional cardiac KO of the α_1A_-AR also had more pathological injury from myocardial infarction after left anterior descending ligation (Yeh et al., 2017; Zhang et al., 2020). Together, these results strongly suggest that the α_1A_-AR subtype mediates ischemic protection in the heart.

### Hypertension

α_1_-ARs are highly expressed in vascular smooth muscle (Hussain and Marshall, 1997; Martí et al., 2005). The rise in calcium upon stimulation of α_1_-ARs in the vasculature activates myosin light chain kinase and actin/myosin cross-bridge formation to induce vascular muscle contraction and increased blood pressure (Somlyo and Somlyo, 2003). The smaller resistance arteries play a more important role in blood pressure regulation and are under stronger control from the sympathetic nervous system. Signals mediated through α_1_-AR activation have been shown to be involved in blood pressure regulation through their control of calcium release and sensitization and signaling through mechanisms involving PKC, PI3K, Rho Kinase, and MAPK (Woo and Lee, 1999; Wier and Morgan, 2003; Villalba et al., 2007; Gutiérrez et al., 2019).

While α_1_-AR antagonists are effective blockers to treat hypertension, they are used as a second line of defense (Chobanian et al., 2003) because of the side effects, poorer outcomes, and worsening or increased risk of heart failure (ALLHAT Collaborative Research Group, 2000). Using KO mice, the α_1A_ was found to decrease blood pressure upon deletion, but only by 15% of the full phenylephrine effect (Rokosh and Simpson, 2002; Table 2). However, the α_1B_-AR KO mediated 45% of the phenylephrine response (Cavalli et al., 1997; Vecchione et al., 2002). Similar minor effects on blood pressure were observed in the α_1D_-AR KO compared to the α_1A_-AR or α_1B_-AR KOs (Cavalli et al., 1997; Hosoda et al., 2005). Only the α_1D_-AR KO decreased basal resting levels of blood pressure (Vecchione et al., 2002; Hosoda et al., 2005).

Since all of the α_1_-ARs appear to regulate blood pressure to a certain degree, specific blockage of the α_1D_-AR may provide better therapeutics to treat hypertension with less overall side effects on other organ systems. This is because the α_1B_-AR appears to have the strongest effect on blood pressure while α_1D_-AR blockage would still lower blood pressure but is not expressed or minimally expressed in the heart (Price et al., 1994; Scofield et al., 1995) or the brain (Tanoue et al., 2002; Sadalge et al., 2003), thereby reducing potential side effects. The α_1D_-AR is also expressed and regulates contraction in the small resistance mesenteric beds which is an important contributor to total peripheral resistance (Christensen and Mulvany, 1993; Hrometz et al., 1999; Gisbert et al., 2002; Methven et al., 2009). The α_1B_-AR subtype controls the neuroeffector junction and sympathetic regulation of the baroreflex response (Townsend et al., 2004) and both the α_1A_- and α_1B_-AR subtypes regulate physiological hypertrophy (O’Connell et al., 2003). The α_1A_-AR as reviewed above is a major regulator of neurotransmission and cognition; thus, blockage of α_1A_- or α_1B_-ARs would affect more off targets than vascular smooth muscle. Therefore, antagonists against the α_1D_-AR subtype might be more effective therapeutically against hypertension by avoiding negative side effects on the heart and brain but may focus effects better on blood pressure regulation.

## Metabolism

The sympathetic nervous system is known to regulate many aspects of metabolism. α_1_-ARs stimulation has long been known to regulate gluconeogenesis in the liver (Chan and Exton, 1978; Hue et al., 1978; García-Sáinz and Hernández-Sotomayor, 1985; de Oliveira et al., 2013). α_1_-ARs also regulate somatostatin-induced gluconeogenesis in the kidney (Dileepan et al., 1982; Dileepan and Wagle, 1985). Gluconeogenesis generates the synthesis of glucose from non-carbohydrate sources while glycolysis breaks down glucose to yield energy (i.e., ATP). Gluconeogenesis becomes important during fasting or starvation when glucose is needed by the cell after glycogen is depleted. α_1_-AR agonists also stimulate glycogen phosphorylase activity, the rate limiting step in glycogen breakdown, which inhibits glycogen synthesis, and increases the breakdown of glycogen (Assimacopoulos-Jeannet et al., 1977; Aggerbeck et al., 1980; Thomas et al., 1985; Ballou et al., 2001; de Oliveira et al., 2013) and stimulates the release of glucagon from the pancreas (Ahrén and Lundquist, 1987; Skoglund et al., 1987; Vieira et al., 2004). However, recent studies have indicated that α_1_-ARs regulate metabolism at a much more systemic level as reviewed below.

### α_1_-AR Stimulation Increases Glucose Tolerance

α_1_-AR stimulation is known to increase glucose uptake in the heart or in primary myocytes (Doenst and Taegtmeyer, 1999; Egert et al., 1999; Shi et al., 2016, 2017; Sato et al., 2018; Papay and Perez, 2020). The systemically expressing CAM α_1A_ but not the CAM α_1B_-AR mice increased glucose uptake into the heart and only the α_1A_-AR KO mice displayed decreased glucose uptake into the heart (Shi et al., 2017). In corroboration, the α_1A_-selective agonist, A61603 increased glucose uptake into primary cardiomyocytes or human α_1A_-AR transfected Chinese hamster ovary (CHO) cells (Sato et al., 2018). While glucose uptake into the heart appears α_1A_-AR specific, both the α_1A_- and α_1B_-AR subtypes mediate glucose uptake into other tissues. The systemically expressing CAM α_1A_ and α_1B_-AR mice both increased glucose uptake into adipose tissue and skeletal muscle while KO of the respective subtype decreased glucose uptake into those same tissues (Shi et al., 2017). The mechanism of α_1A_-AR mediated glucose uptake in the myocyte was through PKCδ signaling that resulted in GLUT 1/4 translocation which causes their activation to transport glucose into the cell (Shi et al., 2016).

The KO and CAM mice also displayed effects on glucose utilization and homeostasis. Both the systemically expressing CAM α_1A_- and α_1B_-AR mice had an increased tolerance for glucose, lower fasting glucose levels while KO mice had poor tolerance and high blood glucose after fasting (Shi et al., 2017). α_1_-AR stimulation also increased glucose absorption in the intestines (Mourad and Saadé, 2011). Hypothalamic central administration of prazosin increased plasma glucose levels (Murashita et al., 2007; Ikegami et al., 2013b) and glucose intolerance (Ikegami et al., 2013a). When fatty acid oxidation was suppressed centrally in the brain, α_1_-ARs stimulated the counter-regulatory increases in plasma glucose levels (Sajapitak et al., 2008). A metabolomic analysis in a neuronal cell culture also showed that α_1_-AR stimulation results in lower levels of carbohydrates (Wenner et al., 2016). These results are consistent with other studies in the α_1B_-AR KO mice which displayed insulin resistance and dysfunctional glucose homeostasis (Burcelin et al., 2004) and the use of prazosin treatment, an α_1_-AR antagonist, which increases risk of metabolic syndrome and high fasting plasma glucose levels in patients with benign prostatic hyperplasia (Lee et al., 2013). The mechanism of the increase in glucose tolerance and lowering of plasma glucose levels is likely due to the increased utilization of glucose through uptake and oxidation in various organs.

### α_1_-AR Mediated Glucose Oxidation in the Heart

α_1_-AR stimulation can also directly increase glucose oxidation in both normal and ischemic primary adult myocytes performed by measuring the rate of ^14^C-CO_2_ production using ^14^C-glucose as a substrate (Papay and Perez, 2020). This study confirmed that the glucose uptake into the heart also drives the oxidation of glucose for energy utilization to the heart. Stimulation of glucose oxidation in the heart improves the recovery from damage during ischemia (Dyck et al., 2006; Ussher et al., 2012; Masoud et al., 2014; Li Y. et al., 2017). Ischemia in the heart can increase glucose uptake by increasing the translocation of GLUT 1/4 (Egert et al., 1999), as this was also shown to be mediated by the α_1A_-AR (Shi et al., 2016). The α_1_-AR mediated glucose oxidation in primary myocytes was also blocked by PKC and AMPK inhibitors (Papay and Perez, 2020) consistent with the role of PKCδ in translocating the glucose transporters in the heart by the α_1A_-AR (Shi et al., 2016). α_1_-AR stimulation increased glucose uptake in the L6 skeletal muscle cell line also through an AMPK pathway (Hutchinson and Bengtsson, 2006). AMPK is an energy sensor that can regulate the rate of glucose and fatty acid uptake and oxidation according to the needs of the cell. AMPK signaling is cardioprotective during heart failure by switching the energy production in the heart from fatty acid oxidation to glucose oxidation (Kim et al., 2012). AMPK also can increase glucose uptake during ischemia to prevent post-ischemic cardiac damage and dysfunction (Russell et al., 2004; Kim et al., 2011). While α_1A_-AR mediated ischemic preconditioning was mediated through PKC (Rorabaugh et al., 2005), PKC was also shown to mediate its protection against ischemic damage through AMPK (Wang et al., 2011). These results suggest that glucose uptake and subsequent oxidation in the heart may be α_1A_-AR specific, signal through PKC/AMPK activation and may mediate α_1A_-AR’s cardioprotective effects during ischemia and heart failure.

### α_1_-AR Mediated Glucose Metabolism in Other Tissues

α_1_-ARs are the main receptors that regulate the control of hepatic glucose metabolism in mice (Chu et al., 2000; Miyamoto et al., 2012; de Oliveira et al., 2013). α_1_-AR stimulation increased glucose uptake into L6 muscle cells (Hutchinson and Bengtsson, 2005, 2006) and C2C12 skeletal myoblasts (Liu et al., 2001). α_1_-AR stimulation also increases glucose uptake into brown and white adipocytes (Faintrenie and Géloën, 1998; Cheng et al., 2000; Boschmann et al., 2002; Flechtner-Mors et al., 2002, 2004; Chernogubova et al., 2005). The sympathetic nervous system enhances glucose uptake into human adipocytes independently of insulin action through the α_1_-AR (Flechtner-Mors et al., 2002, 2004; McCarty, 2004). In obese people that have insulin resistance, α_1_-AR stimulation may provide a critical alternative pathway for glucose uptake.

### α_1_-ARs Mediated Fatty Acid Oxidation

The KO and transgenic mice of the α_1_-AR subtypes were used to discern effects of the specific subtypes on general whole-body metabolism. Systemically expressing CAM mice were assessed by indirect calorimetry and found that both CAM α_1A_- and α_1B_-AR mice decreased the respiratory exchange ratio (RER) (ratio of CO_2_ production and O_2_ consumption) which indicated an increase in whole body preference to metabolize fatty acids as a substrate (i.e., fatty acid oxidation) while the KO mice from both subtypes preferred to burn carbohydrates and increased the RER (Shi et al., 2017). It is likely that α_1_-AR stimulation increases fatty acid oxidation in the skeletal muscle as that muscle utilizes 40–50% of a body’s whole energy metabolism. While there is a report that prazosin can increase angiogenesis in skeletal muscle resulting in increased capillarization to improve the diffusion of glucose into the muscle and may increase glucose oxidation due to substrate availability (Akerstrom et al., 2014), prazosin’s effect was due to improved blood flow and not to GLUT 1/4 translocation.

Both systemically expressing CAM α_1A_- and α_1B_-AR mice displayed increased plasma levels of leptin while KO mice decreased leptin levels (Shi et al., 2017). In obese humans, α_1_-AR blockade reduces leptin levels (Ihara et al., 2006). While leptin can also directly increase glucose oxidation in the absence of insulin in skeletal muscle through a neural hypothalamic β-AR mechanism (Nevzorova et al., 2006; Glund et al., 2007; Shiuchi et al., 2009; Minokoshi et al., 2012; Cadaret et al., 2017), leptin mainly increases fatty acid oxidation in skeletal muscle and the liver through α_1_-AR stimulation of AMPK activity (Minokoshi et al., 2002, 2012; Miyamoto et al., 2012).

α_1_-ARs can also couple to peroxisome proliferator-activated receptor-delta (PPARs) to regulate fatty acid oxidation and utilization (Tanaka et al., 2003). PPAR subtypes β/δ are nuclear receptors and serve as sensors of fatty acid levels. They bind and are activated by fatty acids and their derivatives and activate transcription factors to regulate metabolism (Poulsen et al., 2012). Using midodrine to non-selectively stimulate α_1_-ARs, α_1_-ARs activated PPARs and AMPK to increase oxidative phosphorylation in rat skeletal muscle or in C2C12 skeletal muscle cells (Lee et al., 2020). PPARs are crucial to maintain normal cardiac function and its energy requirements. Cardiac-targeted KO of PPARδ decreases basal fatty acid oxidation leading to cardiac dysfunction, lipid accumulation and heart failure (Cheng et al., 2004). Overexpression of a CAM PPARβ/δ leads to increased levels of fatty acid oxidation (Barak et al., 2002).

Tissue transglutaminase (TG2) is an ubiquitous and multi-functional protein and enzyme with regulatory crosslinking functions in cell adhesion and the cytoskeleton but also has GTP hydrolyzing activities (Fesus and Piacentini, 2002; Eckert et al., 2014). Phenylephrine, an α_1_-AR non-selective agonist was injected into TG2 KO mice and resulted in a lowering of the RER indicating that the mice were burning more whole-body fatty acids than glucose when compared to normal mice with intact TG2 (Lénárt et al., 2020). α_1_-AR stimulation also resulted in lower organ damage particularly in the heart but also in the lung, liver, kidney, and skeletal muscle and a weaker vasoconstriction response compared to normal mice (Lénárt et al., 2020). When the same mice were given a β_3_-AR agonist, the RER was lowered and organ damage was changed to the same extent in both TG2 KO or normal mice (Lénárt et al., 2020). A β_3_ agonist lowers the RER because of its high density in adipose tissue (Ferrer-Lorente et al., 2005). These results concur with the whole-body indirect calorimetry studies that showed that the systemically expressing CAM α_1_-AR mice burned more fatty acids (Shi et al., 2017) and protected the heart from ischemic damage (Rorabaugh et al., 2005; Shi et al., 2016). TG2 is a protein ubiquitously found in cells and can function in both protein cross-linking and bind GTP to act as a G-protein transducer at α_1_-ARs (Nakaoka et al., 1994; Baek et al., 1996; Feng et al., 1996; Kang et al., 2004).

α_1_-AR stimulation can increase the rate of lipolysis in obese individuals (Flechtner-Mors et al., 2002) increasing the availability of fatty acids. α_1_-ARs stimulation also increase fatty acid oxidation in the liver or in hepatocytes (Sugden et al., 1980; Kosugi et al., 1983; Oberhaensli et al., 1985; de Oliveira et al., 2013) and during a high-fat diet can reduce hepatic steatosis (i.e., fatty liver disease) (Nakade et al., 2020). Using a metabolomic analysis, the α_1A_-AR selective agonist, A61603, produced a reduction in cardiac polyunsaturated fatty acids (Willis et al., 2016). The systemically expressed CAM α_1A_-AR mice displayed significantly decreased fasting plasma triglycerides while α_1A_-AR KO displayed increased levels of triglycerides (Shi et al., 2017). In contrast, α_1_-AR blockers such as prazosin or doxazosin have been reported to lower triglycerides and cholesterol but increase high density lipoproteins in humans (Ferrara et al., 1986; Weinberger, 1986; Trost et al., 1987). The reason for this discordance is unknown. However, α_1_-AR quinazoline-based antagonists and particularly prazosin and doxazosin have known non-α_1_-AR mediated off-target effects (Benning and Kyprianou, 2002; Lin et al., 2007; Isgor and Isgor, 2012).

## Pharmacological Interventions

### Development of α_1_-AR Subtype-Selective Ligands

Development of selective α_1_-AR subtype ligands has not been a focus in the pharmaceutical industry because of the ALLHAT Collaborative Research Group (2000) clinical trials and the major cardiovascular events that occur when α_1_-AR antagonists are used. There are still no selective blockers or agonists for the α_1B_-AR, and while BMY 7378 is somewhat selective for the α_1D_-AR (Goetz et al., 1995), there is no clear clinical target. α_1A_-AR antagonists have fared better in drug development because they target prostate and lower urinary tract problems which often affect men with increasing age and who also have high blood pressure; thus, tackling two problems with one therapeutic (Van Asseldonk et al., 2015). However, these therapeutics, as are all α_1_-AR antagonists, are contraindicated in people with heart problems (O’Connell et al., 2013). Recent studies also suggest that α_1_-AR antagonists increase mortality rates in hospitalized patients with Covid-19 (Rose et al., 2020).

**FIGURE 1:**
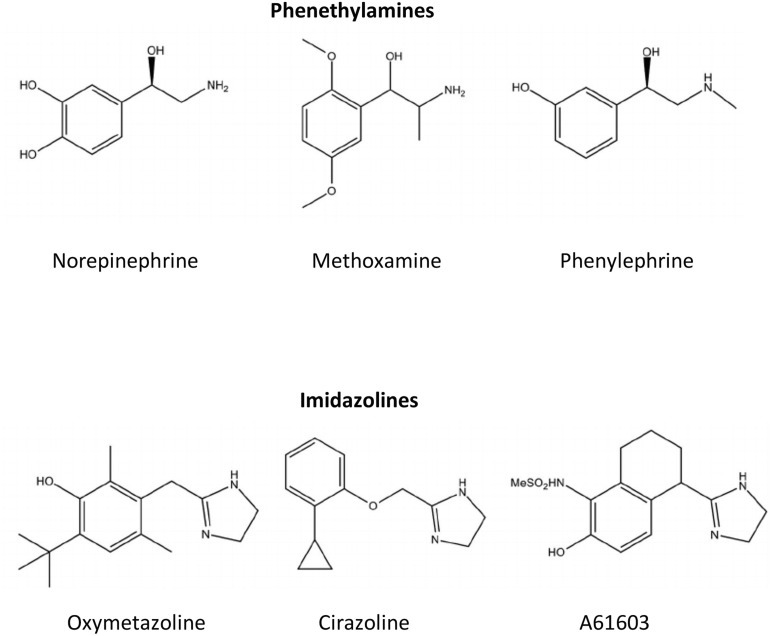
Chemical structures for common α_1_-AR agonists. Ligands such as norepinephrine that contain a phenyl, two-carbon ethyl, and amine group in their structural backbone are referred to as phenethylamines. Ligands that contain an imidazole ring in its structural backbone instead of the phenyl ring are referred to as imidazolines.

The above review indicates that the α_1A_-AR subtype may be a target for drug development for cardioprotection and cognitive enhancement in dementia-type diseases. The potential for α_1A_-AR agonists to be used to treat these diseases has a major problematic side effect of increasing blood pressure (Woo and Lee, 1999; Wier and Morgan, 2003; Villalba et al., 2007; Gutiérrez et al., 2019). This drawback has limited the development of α_1_-AR-based therapeutics by pharmaceutical companies (Fordyce et al., 2015). However, there are two avenues of development that are recently being used to circumvent the blood pressure effect of α_1A_-AR agonists. The first one is the use of the imidazoline pharmacophore instead of the endogenous phenethylamine pharmacophore that is possessed by norepinephrine, epinephrine and several other α_1_-AR agonists (Figure 1).

### Imidazolines

In general, imidazolines have better binding and functional agonistic selectivity for α_2_-ARs and reduce blood pressure by decreasing norepinephrine release at the α_2A_-AR autoreceptor (Ruffolo et al., 1983). However, in the early days of α_1_-AR agonist drug development, it was noted imidazolines interacted with the α_1_-ARs in a different way structurally than with α_2_-ARs. The Easson-Stedman hypothesis states that adrenergic agonists that are chiral by possessing an asymmetric hydroxyl-substituted benzylic carbon atom will have higher binding affinity and potency for the R(–) (i.e., right hand) isomer when compared to the S(+) (i.e., desoxy) isomer (Easson and Stedman, 1933). Imidazoline binding to α_1_-ARs did not adhere to the Easson-Stedman hypothesis that held with phenethylamines, such as norepinephrine (Patil et al., 1974; Ruffolo et al., 1980, 1983; Hieble et al., 1986). While most imidazolines that selectively bind to the α_2_-AR are agonists, they become weak antagonists at the α_1_-AR (Ruffolo and Waddell, 1982). During drug development, specific substitutions off the imidazoline pharmacophore can convert imidazolines from α_2_-AR agonists to α_1_-AR agonists (Ruffolo et al., 1980; Hieble et al., 1986; Knepper et al., 1995). Furthermore, subsequent studies indicated that imidazolines that had higher affinity for the α_1_-AR than the α_2_-AR had agonist-selectivity for the α_1A_-AR subtype in both binding affinity and function compared to the other two α_1_-AR subtypes, the α_1B_- or α_1D_-AR (Minneman et al., 1994). Structure-function analysis revealed that imidazolines, while agonists at the α_1A_-AR, interact with amino acid residues closer to the cell surface in the α_1A_-AR binding pocket, similar to α_1_-AR antagonists, confirming the differences seen with the Easson-Stedman hypothesis (Waugh et al., 2001). These differences in binding also explained why most imidazolines are partial and not full agonists at the α_1_-ARs.

There are several commercially available imidazolines, such as cirazoline and A61603, that are selective for the α_1A_-AR versus the α_1B_- and α_1D_-AR subtypes and with lower affinity against the α_2_-AR. An analog of cirazoline and an α_1A_-AR partial agonist, RO 115–1240 and later by the commercial product dabuzalgron, was shown to reduce stress urinary incontinence without increasing blood pressure (Blue et al., 2004; Musselman et al., 2004). The therapeutic index is wide enough that R0 115–1240 can contract bladder smooth muscle at a much lower dose than required to contract vascular smooth muscle by the α_1A_-AR. This is possible because of the higher receptor density of the α_1A_-AR in the urinary tract compared with vascular smooth muscle and its partial agonist activity that allows reflex mechanisms to control changes in blood pressure (Ford et al., 1996; Walden et al., 1997; Kava et al., 1998; Musselman et al., 2004; Michel and Vrydag, 2006). While all of the above are indeed possible mechanisms for α_1A_-AR agonists to avoid increasing blood pressure, imidazolines were subsequently shown to have bias signaling or agonist trafficking which can lead to lower efficacy of the signaling pathways known to increase blood pressure. Imidazolines induce a more robust cAMP signaling response versus the inositol phosphate signal which increases calcium release to cause the vascular smooth muscle contraction (Evans et al., 2011; da Silva et al., 2017). Confirming the role of α_1A_-AR-selective imidazolines in cardioprotection, dabuzalgron was shown to protect against cardiac damage induced by doxorubicin (Beak et al., 2017; Montgomery et al., 2017) and A61603 increased inotropy in a mouse model of right heart failure (Cowley et al., 2015), but blood pressure was not assessed at the dosage used in these experiments. Confirming the role of α_1A_-AR-selective imidazolines in enhancing cognition, cirazoline, which crosses the blood brain barrier, was shown to increase cognition in normal mice (Doze et al., 2011).

### Allosteric Modulators

A second avenue of drug development for α_1A_-AR agonists with a wide therapeutic index to avoid increases in blood pressure are allosteric modulators. Allosteric modulators offer greater selectivity in both binding and signaling than conventional ligands which bind to the natural endogenous site on the receptor (i.e., orthosteric) (Christopoulos, 2002). Besides greater selectivity because they bind in a different place than orthosteric agonists that is non-conserved between subtypes of the receptor, allosteric modulators offer many other benefits in therapeutics. These are the saturability of its binding site (i.e., ceiling effect) and conformational or probe bias that can alter the receptor to induce a bias in signaling and activation properties but only when the receptor is already occupied with a specific ligand or probe (Christopoulos, 2002).

Allosteric modulators are classified by their ability to modulate function. Positive allosteric modulators (PAMs) increase a receptor’s functional response while negative allosteric modulators (NAMs) decrease the functional response. There are also neutral or silent allosteric modulators (SAMs) that bind to the receptor and display no measurable changes in function (Lindsley et al., 2016) but can block the effects of PAMs or NAMS (Rodriguez et al., 2005). There are now many GPCR allosteric modulators that have been developed (Chen et al., 2008; Wold et al., 2019; Zhou and Cunningham, 2019; Fasciani et al., 2020) with several in clinical trials or with FDA approval (Wold et al., 2019). The HIV entry inhibitor maraviroc is the most known clinically used GPCR allosteric modulator against the CCR5 receptor (Maeda et al., 2012).

There are a few NAMs that have been characterized for the α_1_-AR but have not been developed for clinical use (Leppik et al., 2000; Sharpe et al., 2003; Chen et al., 2004; Lima et al., 2005; Ragnarsson et al., 2013; Campbell et al., 2017). We have developed the first PAM of the α_1_-ARs with selectivity at the α_1A_-AR subtype. It has the imidazoline pharmacophore and can cross the blood brain barrier in sufficient concentration to cause neurological effects without increased blood pressure (Perez, 2021). We have demonstrated its ability to significantly increase LTP in a mouse model of AD along with increases in cognitive behavior using the Barnes maze and fear-conditioning tests. This was achieved using a 10-month dosing scheme and studies are underway to test effects of this compound in a dose-efficacy preclinical trial for 3 months (Perez, 2021).

### Therapeutic Autoantibodies and Vaccines Against α_1_-ARs

There has been recent work in therapeutic vaccines directed at the α_1_-AR subtypes and their roles in hypertension and cardiovascular disease. Autoantibodies against the α_1_-ARs were first found in patients over 20 years ago with severe hypertension (Fu et al., 1994; Luther et al., 1997; Wenzel et al., 2008). A vaccine made against the second extracellular loop of the α_1D_-AR was found to have antagonistic behavior (Li et al., 2019). The vaccine was injected into spontaneously hypertensive rats (SHR) with or without pre-treatment with NG-nitro-L-arginine methyl ester (L-NAME) to generate NO and to reduce blood pressure (Li et al., 2019). This α_1D_-AR vaccine reduced the systolic blood pressure up to 15 mmHg in the SHR group and up to 29 mmHG in the SHR + L-NAME group. This vaccine also prevented cardiac hypertrophy and fibrosis, vascular remodeling, and renal injury even better than compared to treatment with prazosin, suggesting that the antibody has blocking activity. There is one commercially available α_1D_-AR antagonist, BMY7378 (Goetz et al., 1995), but is not sufficiently selective to avoid blocking the other α_1_-AR subtypes for therapeutic use. Because of the unique amino acid sequence used in a non-conserved region of the second extracellular loop of the receptor, vaccines against the α_1D_-AR subtype would be highly selective and avid to regulate the blood pressure response and avoid blocking the other α_1_-AR subtypes.

However, the vast majority of autoantibodies are associated with agonistic activity resulting from a rise in intracellular calcium, and postulated to result in a vasoconstrictive effect (Bkaily et al., 2003; Karczewski et al., 2010; Yan et al., 2014). However, one controlled clinical study indicated that hypertensive patients with α_1_-AR autoantibodies displayed normal cardiovascular responses to α_1_-AR stimulation and removal of α_1_-AR autoantibodies by immunoadsorption did not alter that response (Schroeder et al., 2012).

While the autoantibody against the α_1D_-AR appears antagonistic, several autoantibodies have been developed or discovered against the first or second extracellular loop of the α_1_-AR appear to be agonistic in behavior (Zhou et al., 2008; Karczewski et al., 2012; Hempel et al., 2016; Wallukat et al., 2020). While developing these autoantibodies for cardioprotective effects for the α_1A_-AR may be tempting, they may not be regulated by the normal desensitization and negative feedback mechanisms common in GPCRs to turn off or wane the signal, resulting in abnormal and non-physiological signaling and proliferation (Zhou et al., 2008; Karczewski et al., 2018; Becker et al., 2019; Wallukat et al., 2020). This abnormal signaling and proliferation may account for the vascular damage that many autoantibodies also impart (Zhou et al., 2008; Karczewski et al., 2012, 2018; Becker et al., 2019; Wallukat et al., 2020). Autoantibodies against the α_1_-AR have also been associated with coronary heart disease (Thyrian et al., 2018), cardiac remodeling and dysfunction (Zhou et al., 2005; Li T. et al., 2017), pre-eclampsia (Ma et al., 2013), thromboangiitis obliterans (Buerger’s Disease) (Klein-Weigel et al., 2014), AD and vascular dementia (Karczewski et al., 2012, 2018; Hempel et al., 2016), and prostate cancer (Wallukat et al., 2020). Therefore, both agonistic and antagonistic autoantibodies against the α_1_-AR subtypes would need to be thoroughly analyzed for off target effects.

## Summary

α_1_-ARs are part of the adrenergic family of sympathetic control and have long been known to regulate blood pressure, smooth muscle contraction and cardiac hypertrophy. In recent work, α_1A_-AR stimulation also mediates adaptive effects and signals in the heart that lead to protective outcomes against ischemia and heart failure. They are also highly expressed in the cognitive centers of the brain and stimulation of α_1_-ARs, particularly the α_1A_-AR, can increase both short-term as well as LTP leading to increased learning and memory functions. With its ability to increase adult neurogenesis, there is a potential for α_1A_-AR agonists or positive allosteric modulators to treat AD and to protect the heart at the same time. α_1_-AR stimulation also mediates several aspects of whole-body and organ-specific metabolism to regulate glucose uptake, gluconeogenesis, glucose breakdown, lipolysis, and fatty acid oxidation for energy production. The regulation of cardiac metabolism by the α_1A_-AR is likely a contributing factor for its protective effects in the heart. For pharmacological interventions, it is suggested that therapeutics that focus on α_1A_-AR agonism be developed. To avoid the potential side effects on blood pressure, the imidazoline rather than the phenethylamine pharmacophore should be of primary focus for drug discovery. Several α_1__A_-AR imidazoline-based agonists have been used in preclinical studies and allosteric agonists that will not increase blood pressure are now in development for heart failure and AD.

## Author Contributions

DP performed all of the research, writing, and editing of this manuscript.

## Conflict of Interest

The author declares that the research was conducted in the absence of any commercial or financial relationships that could be construed as a potential conflict of interest.

## Footnotes

1 The α_1C_-AR was first designated novel but more detailed analysis revealed that it was a bovine analog of the α_1A_-AR (Schwinn et al., 1990; Laz et al., 1994; Perez et al., 1994). To avoid confusion, an actual novel α_1_-AR subtype discovered through molecular cloning was designated the α_1D_-AR (Perez et al., 1991) and the α_1C_ designation was dropped from the classification scheme.
